# Effects of low-load resistance training with blood flow restriction on muscle fiber myofibrillar and extracellular area

**DOI:** 10.3389/fphys.2024.1368646

**Published:** 2024-02-20

**Authors:** Cleiton A. Libardi, Joshua S. Godwin, Tanner M. Reece, Carlos Ugrinowitsch, Trent J. Herda, Michael D. Roberts

**Affiliations:** ^1^ MUSCULAB–Laboratory of Neuromuscular Adaptations to Resistance Training, Department of Physical Education, Federal University of Sao Carlos, Sao Carlos, Brazil; ^2^ School of Kinesiology, Auburn University, Auburn, AL, United States; ^3^ Program in Physical Therapy, Washington University School of Medicine, St. Louis, MO, United States; ^4^ School of Physical Education and Sport, University of Sao Paulo, Sao Paulo, Brazil; ^5^ Department of Health Sciences and Human Performance, The University of Tampa, Tampa, FL, United States; ^6^ Department of Health, Sport, and Exercise Sciences, University of Kansas, Lawrence, KS, United States

**Keywords:** vascular occlusion, myofibers, myofibrils, extracellular matrix, exercise

## Abstract

Blood flow restriction applied during low-load resistance training (LL-BFR) induces a similar increase in the cross-sectional area of muscle fibers (fCSA) compared to traditional high-load resistance training (HL-RT). However, it is unclear whether LL-BFR leads to differential changes in myofibrillar spacing in muscle fibers and/or extracellular area compared to HL-RT. Therefore, this study aimed to investigate whether the hypertrophy of type I and II fibers induced by LL-BFR or HL-RT is accompanied by differential changes in myofibrillar and non-myofibrillar areas. In addition, we examined if extracellular spacing was differentially affected between these two training protocols. Twenty recreationally active participants were assigned to LL-BFR or HL-RT groups and underwent a 6-week training program. Muscle biopsies were taken before and after the training period. The fCSA of type I and II fibers, the area occupied by myofibrillar and non-myofibrillar components, and extracellular spacing were analyzed using immunohistochemistry techniques. Despite the significant increase in type II and mean (type I + II) fCSA (*p* < 0.05), there were no significant changes in the proportionality of the myofibrillar and non-myofibrillar areas [∼86% and ∼14%, respectively (*p* > 0.05)], indicating that initial adaptations to LL-BFR are primarily characterized by conventional hypertrophy rather than disproportionate non-myofibrillar expansion. Additionally, extracellular spacing was not significantly altered between protocols. In summary, our study reveals that LL-BFR, like HL-RT, induces skeletal muscle hypertrophy with proportional changes in the areas occupied by myofibrillar, non-myofibrillar, and extracellular components.

## Introduction

Resistance training has been extensively documented to increase cellular and tissue-level hypertrophy in skeletal muscle (Roberts et al., 2023). Blood flow restriction applied during low-load resistance training (LL-BFR) is an effective strategy to increase the cross-sectional area of muscle fibers (fCSA) (i.e., muscle hypertrophy) (Patterson et al., 2019). Recently, we demonstrated that LL-BFR induces similar hypertrophy of type I and II fibers compared to traditional high-load resistance training (HL-RT), regardless of sex (Reece et al., 2023). Although muscle fiber hypertrophy is similar between LL-BFR and HL-RT, the differential expansion of the myofibrillar (e.g., myosin heavy chain and light chain isoforms, various actin isoforms, z-line proteins, etc.) and non-myofibrillar components (e.g., intracellular fluids, sarcoplasmic proteins and enzymes, glycogen, mitochondria, etc.) may be influenced by these different training protocols. Moreover, no study has aimed to examine if myofiber spacing is differentially affected by these training protocols.

There has been a renewed interest in examining myofibrillar adaptations to resistance training (Jorgenson et al., 2020; Roberts et al., 2020; Roberts et al., 2023). MacDougall et al. (MacDougall et al., 1982) used electron microscopy investigation to show that longer-term resistance training disproportionately increases sarcoplasmic spacing. Contrary to this finding, more recent evidence suggests traditional HL-RT typically induces a proportional expansion in the myofibrillar and non-myofibrillar areas of muscle fibers (Wang et al., 1993; Fox et al., 2021; Ruple et al., 2021; Vann et al., 2022). Additionally, an increase in myofibrillar number, but not size, has been shown to accompanies the increases in fCSA with weeks of resistance training (Jorgenson et al., 2023). However, select evidence suggests that non-myofibrillar area expansion may predominate during muscle fiber hypertrophy in resistance training protocols that diverge from traditional methods (e.g., an unconventional or very high-volume training paradigms) (Haun et al., 2019; Fox et al., 2021; Vann et al., 2022). Despite extensive research into these intracellular changes in recent years, little is known regarding how LL-BFR affects these features in muscle fibers.

To the best of our knowledge, only one study has explored the effects of LL-BFR on intracellular myofibril content. Wilburn et al. (2021) conducted a case study composed by a single exercise session in which one of the subject’s legs underwent LL-BFR, while the contralateral leg was subjected to HL-RT. Vastus lateralis muscle biopsies were obtained before and 30 min after each exercise protocol. The primary findings indicated that the LL-BFR protocol induced acute myofibrillar alterations in a wave-like pattern, likely attributed to the pouches formed by fluid accumulation in the sarcoplasm, which appeared to disrupt the linear alignment of sarcomeres. Additionally, muscle damage (i.e., Z-disc disruption, thick and thin filament rupture) was not observed to the same extent as with HL-RT. These findings suggest that, at least acutely, fluid accumulation induced by LL-BFR may cause changes in the non-myofibrillar area distinct from HL-RT. However, it remains unknown whether these acute changes demonstrated by Wilburn et al. (2021) are also observed a LL-BFR training period.

Therefore, the aim of the present study was to investigate whether the hypertrophy of type I and II fibers induced by LL-BFR or HL-RT is accompanied by differential changes in myofibrillar and non-myofibrillar areas. We additionally examined if extracellular spacing was differentially affected between these two training paradigms. We hypothesized that there would be a decrease in myofibrillar area and an increase in non-myofibrillar area with type I and II fCSA increases following LL-BFR. In contrast, we hypothesized that HL-RT would promote conventional hypertrophy, consistent with findings from previous studies (Fox et al., 2021; Ruple et al., 2021; Vann et al., 2022). Finally, we hypothesized that extracellular area would be greater following LL-BFR.

## Methods

### Participants

This study was a secondary analysis from a subset of participants from a study that was reviewed and approved by the institutional review board for human subjects (IRB Study no. 00147374). The study conformed to the standards set by the latest revisions of the Declaration of Helsinki and was registered as a clinical trial before the recruitment of the first participant (25 June 2021, NCT04938947). All participants read and signed an informed consent form before the start of the experimental protocol. Ten women (22 ± 5 years old, 71.1 ± 12.3 kg, 166 ± 9.1 cm, 25.8 ± 4.5 kg/m^2^) and 10 men (21.5 ± 2 years old, 78.4 ± 19.7 kg, 179 ± 7.3 cm, 24.4 ± 5.4 kg/m^2^) who were engaged in recreational activities (e.g., jogging and intramural sports), excluding resistance training, were eligible to participate in the study. All participants were free from cardiometabolic diseases or medical conditions that precluded the collection of muscle biopsies.

### Experimental design

More detail regarding the experimental design can be found in our parent publication (Reece et al., 2023). Before resistance training ensued, muscle biopsies were obtained from the vastus lateralis of the right leg. Participants were then assigned to either the LL-BFR (*n* = 11, 5 female) group or the HL-RT (*n* = 9, 5 female) group. Training was performed 3 days a week for 6 weeks. Two to 7 days after the last training session, participants post-intervention muscle biopsies were obtained.

### Muscle biopsies

Participants were positioned supine on a table and given a subcutaneous lidocaine (1%, 1.5 mL) injection above the skeletal muscle fascia at the collection site. After a 5-min wait for the anesthetic to take effect, a precise pilot incision was made using a sterile surgical blade. A 5-gauge Bergstrom biopsy needle, following Evans et al. (1982), was used to extract approximately 50–80 mg of skeletal muscle tissue with suction. Afterward, the harvested tissue was carefully cleaned of residual blood and connective tissue, was preserved in freezing media for histological analysis (Tissue-Tek^®^, Sakura Finetek Inc.; Torrance, CA, United States), frozen in liquid nitrogen-cooled isopentane, and stored at −80°C until shipment to Auburn University for sectioning and histology.

### LL-BFR and HL-RT protocols

Training protocols are described in detail in Reece et al. (2023). Briefly, each session consisted of three sets of bilateral leg extensions performed to the point of muscular failure. The HL-RT consisted of 3 x 8–12 repetitions (∼80% 1RM) with a 2-min rest interval between sets. In the LL-BFR group, the participants performed three sets to failure using a constant load equivalent to 30% of their 1RM with a 1-min rest interval between sets. These participants utilized 10-cm-wide BFR cuffs (SmartTools, Strongsville, OH) applied to the proximal regions of both legs. The cuff pressure was set at 50% of each participant’s estimated arterial occlusion pressure (AOP), determined based on their thigh circumference, ranging from 100 to 180 mmHg (Loenneke et al., 2012). Cuffs remained inflated following specific instructions until the end of the final set of each training session.

### Immunohistochemistry for determining fiber type and phalloidin-actin staining

**FIGURE 1:**
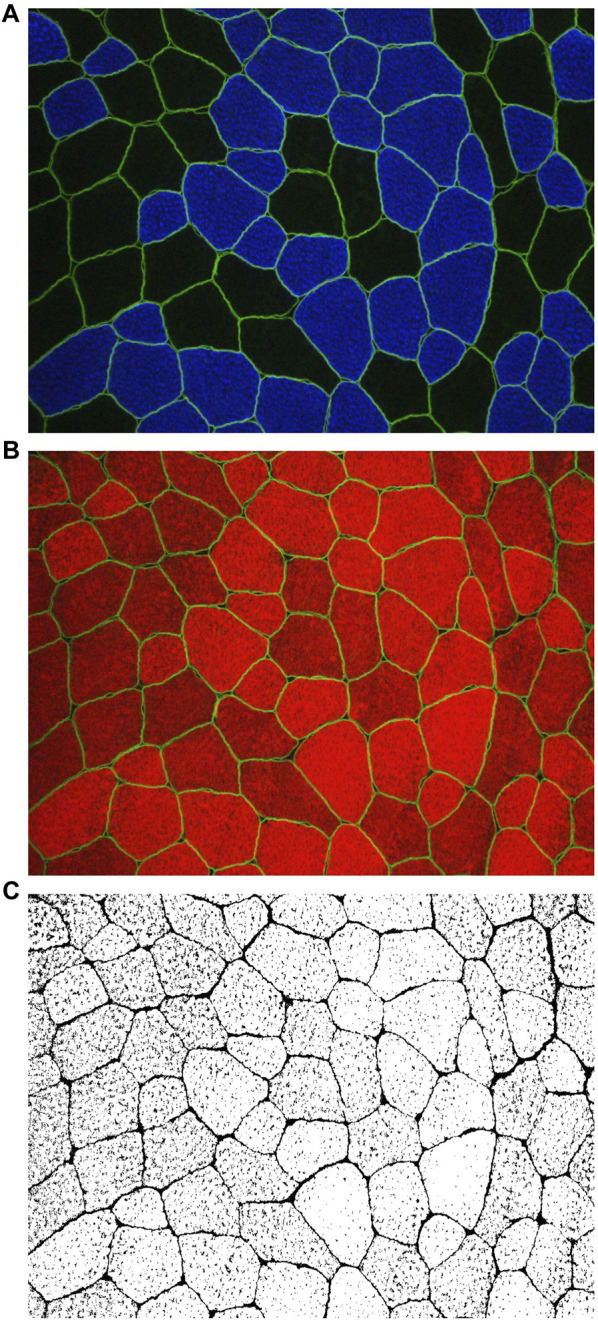
Utilizing images obtained through MyoVision software, the cross-sectional area (fCSA) of both type I and type II muscle fibers was calculated **(A)** Digital images were captured using a fluorescence microscope equipped with a ×20 objective lens (Nikon Instruments, Melville, NY, United States). This staining method facilitated the discrimination of type I fibers, identified by blue cell bodies (detected through the FITC filter), and type II fibers, characterized by unmarked black cell bodies. Additionally, dystrophin was stained bright green. Representative images at ×20 magnification of the phalloidin staining are presented in **(B**, **C)** ImageJ software was employed for image processing, allowing the separation of images into RGB channels. Subsequently, the red channel containing phalloidin staining **(B)** was converted to grayscale for further analysis **(C)**. In **(C)**, the myofibrillar area within myofibers is depicted in white, while non-myofibrillar area is rendered in black.

Initially, the fCSA of types I and II was determined as previously described by our laboratory (Vann et al., 2020). To determine the myofibril area per fiber, F-actin labeling with Alexa Fluor 594-conjugated (AF594) phalloidin was executed in accordance with established protocols as previously described (Gokhin et al., 2008; Duddy et al., 2015; Haun et al., 2019). This staining procedure facilitated the distinction of the sarcolemma (visualized through the FITC filter) and myofibrils (observed via the Texas Red filter). Multiple 20× images section were obtained and the quantification of myofibril area per fiber was conducted utilizing ImageJ software developed by the National Institutes of Health (NIH) according to procedures previously carried out in our laboratory (Fox et al., 2021; Ruple et al., 2021; Michel et al., 2023). In brief, the image scale in ImageJ was set to 0.451 μm per pixel. Images were then separated into RGB channels, and the red channel containing phalloidin staining was converted to grayscale. The threshold function was applied to create a binary black-and-white image distinguishing stained and unstained fiber areas. Individual fibers were then outlined, and myofibril areas were expressed as a percentage of the total fiber area. These values were reported as the percentage of myofibril content within each fiber or as the total contractile protein per fiber (i.e., % myofibril per myofiber × fiber cross-sectional area). Thus, we assumed that changes in proportion of myofibril and non-myofibril of the total muscle fiber cross-section area indicated changes in the areas of the fractions. A visual representation of this image analysis is illustrated in Figure 1 of the results section. To obtain the extracellular area, type I and II fibers were imaged together with their extracellular spaces (intracellular + extracellular area). Then, total area values obtained were subtracted from the fCSA of all fibers. The resultant values were considered extracellular area. The coefficient of variation obtained from the typical error as 0.35% for the intracellular area and 0.0007% for the extracellular area.

### Statistical analyses

A mixed model, assuming group and time as fixed factors and subjects as a random factor, was implemented for the analysis of all variables. In the case of significant *F* values, a Tukey adjustment was used for multiple comparison purposes. The significance level was set at *p* < 0.05. In addition, the effect sizes (ES) and respective confidence intervals (CI) of the differences between the delta change (Post—Pre) of all variables in each group were calculated according to Hedges and Olkin (Hedges and Olkin, 1985). Positive and negative CIs not crossing zero (0) were considered significant (Nakagawa and Cuthill, 2007).

## Results

### Fiber cross-sectional area (fCSA)

**FIGURE 2:**
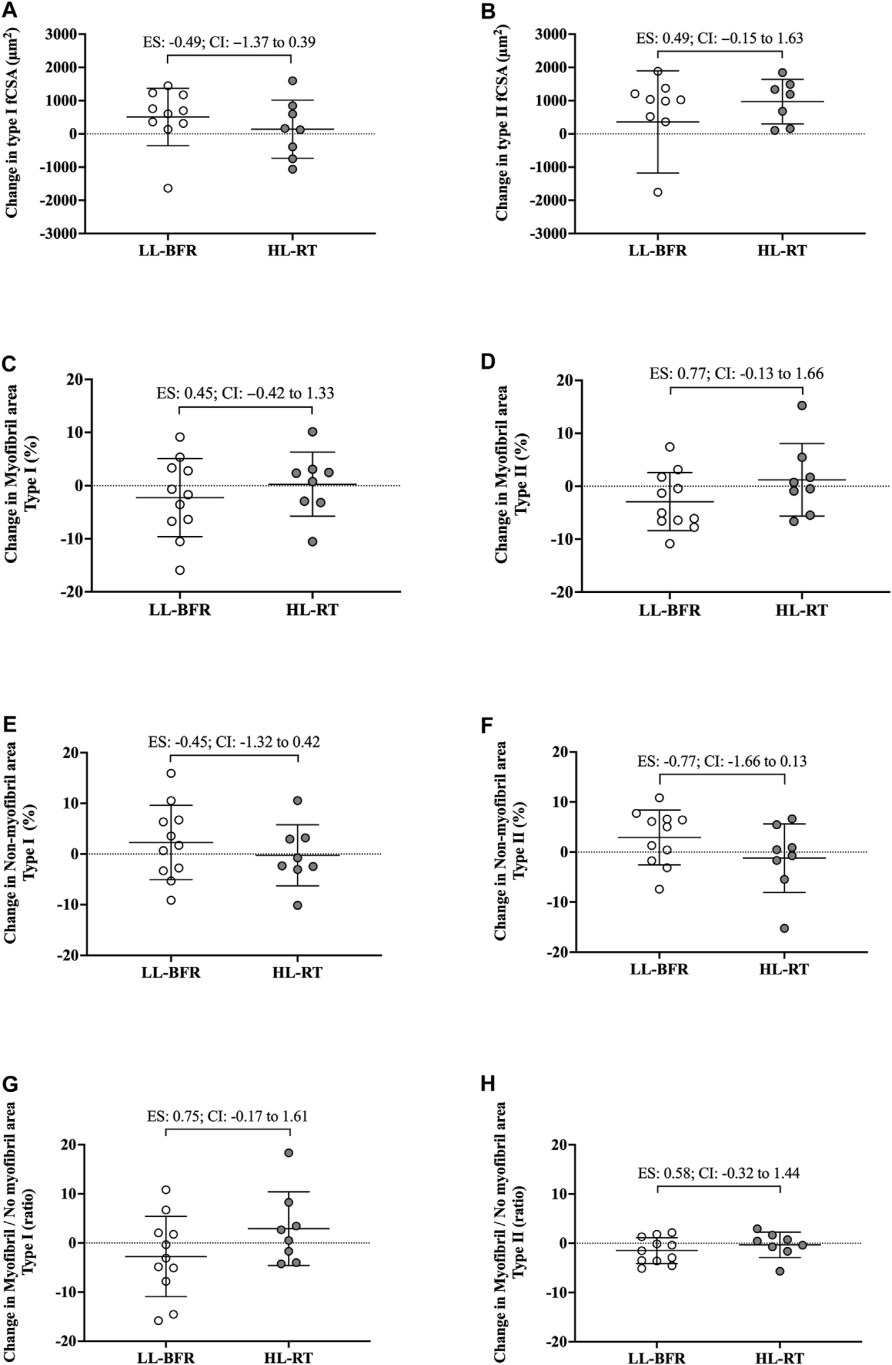
Changes in the cross-sectional area (fCSA), myofibrillar and non-myofibrillar area of type I [**(A, C, E)**, respectively] and type II [**(B, D, F)**, respectively] fibers and the ratio of the myofibrillar and non-myofibrillar area of type I **(G)** and II **(H)** fibers. LL-BFR: blood flow restriction applied during low-load resistance training; HL-RT, high load resistance training; ES, effect size; CI, confidence interval.

**FIGURE 3:**
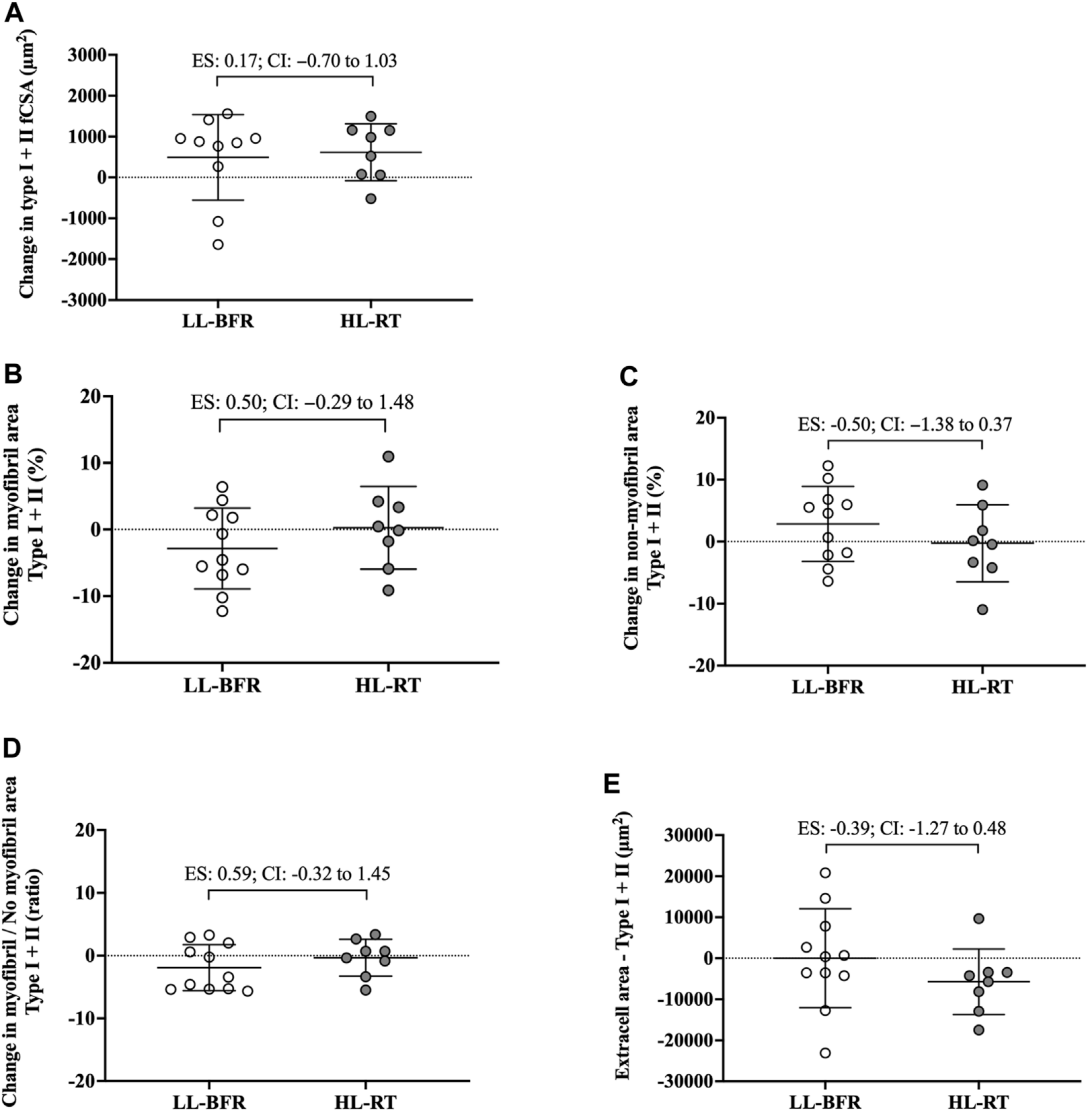
Changes in the cross-sectional area (fCSA), myofibrillar and non-myofibrillar area of type I + II fibers [**(A–C)**, respectively], ratio of the myofibrillar and non-myofibrillar area of fibers type I + II **(D)** and extracellular space of type I + II fibers **(E)**. LL-BFR: blood flow restriction applied during low-load resistance training; HL-RT, high load resistance training; ES, effect size; CI, confidence interval.

There was no significant group vs. time interaction (*F*_[1, 17]_ = 0.14; *p* = 0.71) or group main effect (*F*_[1, 17]_ = 0.17; *p* = 0.68) for mean fCSA. However, a main effect of time was observed (*F*_[1, 17]_ = 7.80; *p* = 0.01). Similarly, only a time effect (*F*_[1, 16.9]_ = 6.02; *p* = 0.02) was observed for type II fCSA, with no significant group vs. time interaction (*F*_[1, 16.9]_ = 1.45; *p* = 0.24) or group main effect (*F*_[1, 17.4]_ = 0.00; *p* = 0.95). For type I fCSA, no effect of group (*F*_[1, 17]_ = 0.22; *p* = 0.64), time (*F*_[1, 17]_ = 2.34; *p* = 0.14) or group vs. time interaction (*F*_[1, 17]_ = 1.15; *p* = 0.29) was observed. Additionally, the 95% CI of ES of the differences between delta changes for type I, type II and mean fCSA indicated no significant differences between groups (Figure 2; Figure 3). The mean and standard deviation values are presented in Table 1.

**TABLE 1:** Cross-sectional area and content of muscle fibers.

Variables (units)	Protocol	Pre	Post	∆
Type I fiber cross-sectional area (μm^2^)	BFR	3,342 ± 926	3,849 ± 1,009	508 ± 865
	HL	3,753 ± 900	3,843 ± 1,268	90 ± 832
Myofibrillar area of type I fibers (%)	BFR	90.01 ± 4.91	87.75 ± 4.93	−2.26 ± 7.35
	HL	87.70 ± 7.21	88.48 ± 6.92	−0.78 ± 5.84
Non-myofibril area of type I fibers (%)	BFR	10.00 ± 4.91	12.25 ± 4.93	2.26 ± 7.35
	HL	12.31 ± 7.21	11.52 ± 6.92	−0.78 ± 5.84
Myofibrillar/Non-myofibril area of type I fibers (ratio)	BFR	11.50 ± 6.14	8.76 ± 4.77	−2.73 ± 8.18
	HL	10.41 ± 7.74	13.50 ± 12.73	3.10 ± 7.03
Type II fiber cross-sectional area (μm^2^)	BFR	3,875 ± 1,740	4,236 ± 917*	361.25 ± 1,538
	HL	3,562 ± 664	4,614 ± 1,094*	1,064 ± 675
Myofibrillar area of type II fibers (%)	BFR	85.61 ± 4.91	82.70 ± 4.31	−2.92 ± 5.48
	HL	83.62 ± 8.71	85.25 ± 5.46	1.63 ± 6.53
Non-myofibril area of type II fibers (%)	BFR	14.40 ± 4.91	17.31 ± 4.31	2.92 ± 5.48
	HL	16.38 ± 8.71	14.75 ± 5.46	−1.63 ± 6.53
Myofibrillar/Non-myofibril area of type II fibers (ratio)	BFR	6.66 ± 2.39	5.17 ± 1.81	−1.49 ± 2.61
	HL	6.60 ± 3.48	6.64 ± 2.76	0.05 ± 2.67
Type I + II fiber cross-sectional area (μm^2^)	BFR	3,599 ± 1,289	4,091 ± 877*	491.49 ± 1,046
	HL	3,697 ± 701	4,339 ± 1069*	642.94 ± 656.30
Myofibril area of type I + II fibers (%)	BFR	87.82 ± 4.61	84.98 ± 4.35	−2.84 ± 6.06
	HL	85.85 ± 7.55	86.60 ± 5.90	0.74 ± 6.00
Non-myofibril area of type I + II fibers (%)	BFR	12.18 ± 4.61	15.02 ± 4.35	2.84 ± 6.06
	HL	14.15 ± 7.55	13.41 ± 5.90	−0.74 ± 6.00
Myofibrillar/Non-myofibril area of type I + II fibers (ratio)	BFR	8.20 ± 3.00	6.30 ± 2.54	−1.92 ± 3.65
	HL	7.86 ± 4.19	7.94 ± 4.02	0.08 ± 3.02
Extra cell area of type I + II fibers (μm^2^)	BFR	14,810 ± 9,372	14,800 ± 8,936	−9 ± 12,060
	HL	15,105 ± 7,947	10,854 ± 6,247	−4,251 ± 8,658

*Significantly different from Pre (time effect, *p* < 0.05).

All data presented as mean ± standard deviation values.

### Myofibrillar and non-myofibrillar areas of muscle fibers

#### Type I fibers

No group vs. time interaction or main effect of group and time was observed for myofibrillar area (*F*_[1, 18]_ = 1.02; *p* = 0.32; *F*_[1, 18]_ = 0.13; *p* = 0.72; *F*_[1, 18]_ = 0.24; *p* = 0.63, respectively), non-myofibrillar area (*F*_[1, 18]_ = 1.02; *p* = 0.32; *F*_[1, 18]_ = 0.13; *p* = 0.72; *F*_[1, 18]_ = 0.24; *p* = 0.63, respectively), and myofibrillar/non-myofibrillar area ratio (*F*_[1, 18]_ = 2.83; *p* = 0.10; *F*_[1, 18]_ = 0.32; *p* = 0.57; *F*_[1, 18]_ = 0.01; *p* = 0.92, respectively). Additionally, the 95% CI of ES of the differences between delta changes for myofibrillar and non-myofibrillar areas for type I fibers indicated no significant differences between groups (Figure 2; Figure 3). The mean and standard deviation values for type I fibers are presented in Table 1.

#### Type II fibers

No group vs. time interaction or main effect of group and time was observed for myofibrillar area (*F*_[1, 18]_ = 2.88; *p* = 0.10; *F*_[1, 18]_ = 0.01; *p* = 0.90; *F*_[1, 18]_ = 0.23; *p* = 0.63, respectively), non-myofibrillar area (*F*_[1, 18]_ = 2.88; *p* = 0.10; *F*_[1, 18]_ = 0.02; *p* = 0.90; *F*_[1, 18]_ = 0.23; *p* = 0.63, respectively), and myofibrillar/non-myofibrillar area ratio (*F*_[1, 18]_ = 1.68; *p* = 0.21; *F*_[1, 18]_ = 0.47; *p* = 0.50; *F*_[1, 18]_ = 1.47; *p* = 0.24, respectively). The mean and standard deviation values for type II fibers are presented in Table 1. The 95% CI of ES analysis revelated no significant differences between groups for changes in myofibrillar and non-myofibrillar areas for type II fibers (Figure 2; Figure 3).

#### Mean fibers (type I + II)

No group vs. time interaction or main effect of group and time was observed for myofibrillar area (*F*_[1, 18]_ = 1.75; *p* = 0.20; *F*_[1, 18]_ = 0.01; *p* = 0.93; *F*_[1, 18]_ = 0.60; *p* = 0.44, respectively), non-myofibrillar area (*F*_[1, 18]_ = 1.94; *p* = 1.75; *F*_[1, 18]_ = 0.01; *p* = 0.93; *F*_[1, 18]_ = 0.60; *p* = 0.44, respectively), myofibrillar/non-myofibrillar area ratio (*F*_[1, 18]_ = 1.73; *p* = 0.20; *F*_[1, 18]_ = 0.23; *p* = 0.63; *F*_[1, 18]_ = 1.46; *p* = 0.24, respectively) and extracellular space (*F*_[1, 18]_ = 0.78; *p* = 0.38; *F*_[1, 18]_ = 0.40; *p* = 0.53; *F*_[1, 18]_ = 0.79; *p* = 0.38, respectively). Additionally, the 95% CI of ES analysis indicated no significant differences between groups for changes in myofibrillar and non-myofibrillar areas and extra cell space for mean fibers (Figure 2; Figure 3). The mean and standard deviation values are presented in Table 1.

## Discussion

In this study, we employed histology techniques to investigate whether LL-BFR or HL-RT induces differential changes in the myofibrillar areas of type I and II muscle fibers and extracellular spacing. Contrary to our initial hypothesis, our findings indicate that myofibrillar and non-myofibrillar areas within muscle fibers exhibited proportional expansion in response to an increase in fCSA, independent of training protocols. Additionally, our data revealed that there was no significant change in the extracellular area surrounding muscle fibers. These observations are the first to show that shorter-term LL-BFR and HL-RT promote similar morphological changes in the muscle tissue of previously untrained males and females.

It has been suggested that skeletal muscle hypertrophy induced by mechanical overload can be attributed mainly to the proportional increase in contractile and non-contractile components of myofibers (Jorgenson et al., 2020; Roberts et al., 2023). This phenomenon has been termed conventional hypertrophy (Roberts et al., 2020). In the present study, the HL-RT group showed an increase of ∼17% in fCSA (type I + II). Notably, the myofibrillar area remained at ∼86%, while the non-myofibrillar area remained at ∼14%. These results are consistent with previous studies that estimated the area occupied by myofibrils to be ∼85% of the intracellular space (MacDougall et al., 1982; Alway et al., 1988; Claassen et al., 1989; Ruple et al., 2021). Furthermore, this proportionality appears to be maintained after an HL-RT program performed for ∼10 weeks as reported in prior research (Fox et al., 2021; Ruple et al., 2021).

Conventional hypertrophy, or the proportional increase in myofibrillar area with an increase in fCSA, has recently been challenged by studies that demonstrated sarcoplasmic expansion after some resistance training protocols. For example, Fox et al. (2021) compared traditional HL-RT with a protocol with frequent manipulation of resistance training variables (e.g., load, sets, muscle action, and rest). In contrast to what was observed after HL-RT, the protocol with variation in exercise stimulus induced an increase in the non-myofibrillar area after 16 resistance training sessions. This phenomenon was also demonstrated by Haun et al. (2019) when conducting a study with a very high volume (i.e., number of sets). Taken together, these findings suggest that unconventional training paradigms appear to induce an expansion of non-myofibrillar components (i.e., sarcoplasm). Based on these observations, as well as the acute promotion of fluid accumulation in the sarcoplasm by BFR (Wilburn et al., 2021), our hypothesis was that LL-BFR training would lead to a disproportionate increase in non-myofibrillar area with hypertrophy of type I and II fibers. However, we observed that both protocols led to conventional hypertrophy, and this could be attributed to several possible explanations. First, there is a lack of agreement between acute and chronic responses after LL-BFR. The absence of studies examining myofibrillar and non-myofibrillar components of skeletal muscle using histological techniques makes it difficult to analyze whether persistent intracellular stability after LL-BFR is a prevalent phenomenon. Despite this, some studies have indirectly investigated fluid shifts using ultrasound. In this regard, greater acute cellular swelling was observed when measuring muscle thickness at multiple time points (0 min, 15 min, 60 min, 180 min, and 48 h) following LL-BFR compared to HL-RT (Farup et al., 2015; Freitas et al., 2017). However, these differences were not observed after 16 training sessions (Farup et al., 2015). As the greater early muscle thickness for LL-BFR is unlikely to be primarily due to increased muscle damage induced by this training protocol (Patterson et al., 2019; Wilburn et al., 2021), it is plausible to suggest that the increase in intracellular or extracellular fluid following sessions may subside over time (Reidy et al., 2017). Second, evidence indicates that sarcoplasmic expansion is more frequently observed in well-trained individuals when they engage in an unconventional training regimen compared to novices (MacDougall et al., 1982; Haun et al., 2019; Fox et al., 2021). There is speculation that a threshold for the accumulation of myofibrillar proteins may develop with years of training (Roberts et al., 2020). As a result, muscle fiber hypertrophy might occur through an increase in non-contractile components, strategically preparing muscle cells for the eventual incorporation of myofibrils (Roberts et al., 2020). Hence, the phenomenon of sarcoplasmic expansion following LL-BFR may be a transient occurrence, or one primarily observed in well-trained individuals. Thus, further research is needed to add clarity in this regard.

There are notable limitations to the current study. First, is the limited number of participants. Additionally, phalloidin staining was used for extrapolating myofibril data. Notably, this technique yields a relatively crude two-dimensional rendering of myofiber characteristics and does not take into consideration three-dimensional features that may have been affected (e.g., myofibril branching) (Willingham et al., 2020). It is important to emphasize that we assessed changes in the proportion of myofibril and non-myofibril within muscle fiber cross-sectional area, and not the actual area of each fraction. Moreover, the Hornberger laboratory recently developed an immunofluorescent myofibril imaging technique using deconvolution (termed FIM-ID) that provides exceptional resolution of myofibril number and sizing (Jorgenson et al., 2023). Given that we did not preserve the current tissue accordingly to perform this technique, future examination using a similar study design and said methods are needed. Finally, the limited 18 training sessions warrants further discussion. Indeed, evidence from past shorter-term interventions suggests that 14–21 training sessions is minimally needed to reliably detect myofiber hypertrophy (Goreham et al., 1999; Damas et al., 2016; Mesquita et al., 2023), and this certainly warrants a longer-term intervention with the current training protocols. However, despite the limited training duration, interesting differences between protocols were evident. First, although the CI of ES crossed zero for all variables analyzed, data in Figure 2B show that pre-to-post intervention change scores in type II fCSA between training paradigms presented a moderate effect (ES: 0.73, CI: −0.10–1.70) whereby values were lower in the LL-BFR protocol *versus* HL-RT protocol (change scores were 19.9% *versus* 29.3%, respectively). Second, data in Figure 2D show that the pre-to-post intervention change scores in type II myofibrillar spacing between training paradigms presented a moderate effect (ES: 0.77, CI: −0.13–1.66) whereby values were lower in the LL-BFR protocol *versus* HL-RT protocol (change scores were −2.92% *versus* 1.63%, respectively). A potential interpretation of these effect sizes is that: 1) longer-term LL-BFR training may be needed to produce comparable type II fiber hypertrophy, and 2) continued type II fiber hypertrophy with LL-BFR may eventually lead to a disproportionate increase in non-myofibrillar spacing. However, given that this speculation is based on moderate effect sizes observed after 18 training sessions with multiple biopsy (acute and chronic) sampling throughout are needed.

## Conclusion

In summary, the novel preliminary data demonstrate that 6 weeks of LL-BFR and HL-RT result in proportional expansion in both myofibrillar and non-myofibrillar areas, contributing to the hypertrophy of type II fibers. Given the stated limitations, longer-duration protocols with more advanced imaging techniques (e.g., three-dimensional SEM or FIM-ID) are needed to fully delineate how LL-BRF training affects cellular morphology.

## Data Availability

The original contributions presented in the study are included in the article/Supplementary Material, further inquiries can be directed to the corresponding authors.
